# Severe hearing loss and outer hair cell death in homozygous Foxo3 knockout mice after moderate noise exposure

**DOI:** 10.1038/s41598-017-01142-3

**Published:** 2017-04-21

**Authors:** Felicia Gilels, Stephen T. Paquette, Holly J. Beaulac, Anwen Bullen, Patricia M. White

**Affiliations:** 1 Department of Neuroscience, University of Rochester School of Medicine and Dentistry, 601 Elmwood Avenue, Rochester, NY 14642 USA; 2 UCL Ear Institute, Faculty of Brain Sciences, University College London, 332 Gray’s Inn Road, London, WC1X 8EE UK; 3 Department of Pathology, University of Rochester School of Medicine and Dentistry, 601 Elmwood Avenue, Rochester, NY 14642 USA; 4 Ortho Clinical Diagnostics, 513 Technology Blvd, Rochester, NY 14626 USA

## Abstract

Noise induced hearing loss (NIHL) is a disease that affects millions of Americans. Identifying genetic pathways that influence recovery from noise exposure is an important step forward in understanding NIHL. The transcription factor Foxo3 integrates the cellular response to oxidative stress and plays a role in extending lifespan in many organisms, including humans. Here we show that Foxo3 is required for auditory function after noise exposure in a mouse model system, measured by ABR. Absent Foxo3, outer hair cells are lost throughout the middle and higher frequencies. SEM reveals persistent damage to some surviving outer hair cell stereocilia. However, DPOAE analysis reveals that some function is preserved in low frequency outer hair cells, despite concomitant profound hearing loss. Inner hair cells, auditory synapses and spiral ganglion neurons are all present after noise exposure in the Foxo3KO/KO fourteen days post noise (DPN). We also report anti-Foxo3 immunofluorescence in adult human outer hair cells. Taken together, these data implicate Foxo3 and its transcriptional targets in outer hair cell survival after noise damage. An additional role for Foxo3 in preserving hearing is likely, as low frequency auditory function is absent in noise exposed Foxo3KO/KOs even though all cells and structures are present.

## Introduction

Noise induced hearing loss (NIHL) presents a significant social burden for millions of Americans^1^. Hearing problems are the most common disability for combat veterans^2,3^, costing over one billion dollars annually in the United States^4^. As NIHL also compromises the ability to distinguish speech in background noise, for service personnel it jeopardizes military readiness^5^ and contributes to social isolation after deployment^6^. There are no therapies to correct NIHL, which has life-long effects.

It is imperative, then, to understand how noise damages the cochlea. Intense low frequency noise can drive the loss of outer hair cells^7^, reducing acoustic amplification and raising thresholds^8^. Damaged outer hair cells have higher levels of nitrotyrosine and lipid peroxidation, implicating oxidative stress^9^. Noise may also drive neurite swelling and eliminate auditory synapses in high-frequency inner hair cells^10^. Some of this damage can be blocked by glutamatergic inhibitors^11^, suggesting that excitotoxic signaling by inner hair cells plays a role.

Recent reports have illuminated certain genetic mechanisms that might influence the extent of damage from NIHL. Single nucleotide polymorphisms in SOD2, CAT, GSTM1, PON2, and NOX3^12^, all oxidative stress genes, have been associated with human hearing susceptibility to NIHL^13^. Pejvakin, the protein underlying DFNB59^14^, is a peroxisome protein that enables outer hair cells to withstand the oxidative stress of the normal hearing environment^15^. Administration of the NAD+ precursor nicotinamide riboside promotes sirtuin activity, and reduces NIHL threshold shifts and neurite degeneration in a Sirt3-dependent manner^16^. Taken together, these results suggest that genetic variation may partly underlie the diverse human susceptibility to NIHL^13^.

Forkhead Box O3 (FOXO3) is a winged helix transcription factor that regulates longevity in multiple species^17–19^ including humans^20^. Many extremely old individuals have a rare haplotype of Foxo3 that leads to its overexpression in muscle tissue^21^. Such individuals experience significantly lower levels of heart disease^22^, explaining their longevity at least in part. FOXO-dependent transcription is generally activated by caloric^23^, mechanical^24^, or oxidative stress^25^. In the heart, FOXO3 drives expression of BNIP3 to promote reversible protein catabolism, counteracting cardiac hypertrophy^26,27^. FOXO3-dependent transcription in other cells has more nuanced effects, e.g. FOXO3 can directly increase the expression of multiple oxidative stress reduction genes in endothelial cells, including Sod2 and Cat^28^. Moreover, FOXO3 integrates the cellular response to oxidative stress in several cell types^29,30^. In neurons, FOXO3 promotes delayed apoptosis after excitotoxic injury^31^ through the activation of Bim transcription^32^. In stroke models, loss of FOXO3 protects neurons and reduces cell death^33^. Strikingly, over-expression of FOXO3 in sympathetic neurons induces apoptosis^34^. Thus, the presence of FOXO3 in tissues is not predictive of its function in a stress response.

We previously reported that FOXO3 is expressed in cochlear sensory hair cells and spiral ganglion neurons^35^. Importantly, non-toxic noise exposure drove FOXO3 nuclear localization, suggesting that FOXO3 might be required for auditory function during noise recovery. Here we test that hypothesis by exposing Foxo3KO/KO mice to a defined noise level that does not cause significant permanent effects in Foxo3+/+ members of their strain (FVB/nJ^36^). We then assess auditory function and alterations of anatomical structures in this animal model.

## Results

### Severe hearing loss after noise exposure for Foxo3KO/KO mice

**Figure 1:**
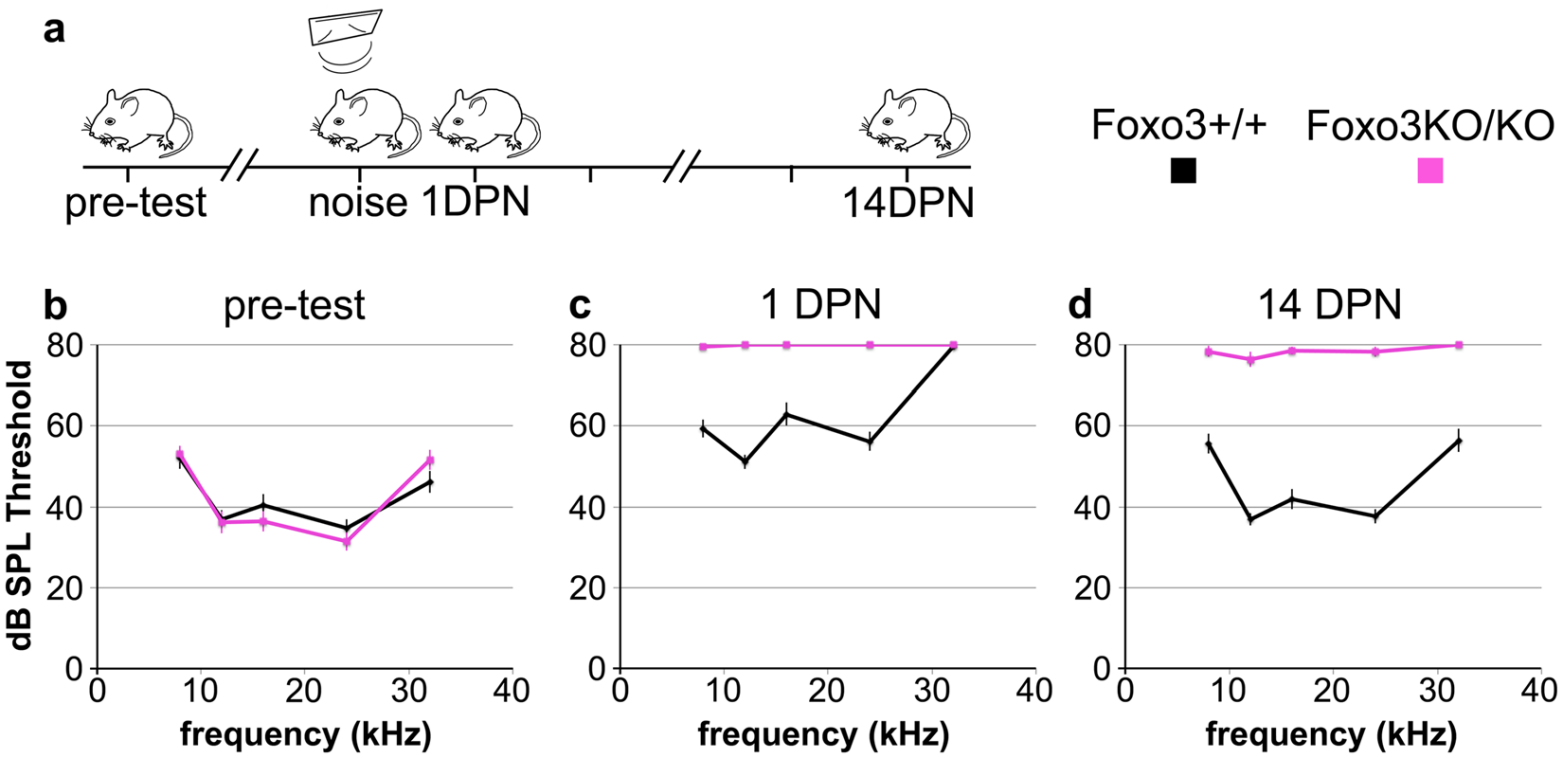
Severe loss of hearing in the Foxo3 KO after noise exposure. (**a**) Schematic of experimental design. Each animal’s hearing thresholds were measured, once prior to noise exposure (“pre-test”) and twice after as depicted, 1 day post noise (DPN) and 14 DPN. (**b**) ABR thresholds were measured at 5 frequencies: 8, 12, 16, 24, and 32 kHz, prior to noise exposure. Black: Foxo3+/+ littermates (n = 17). Pink: Foxo3KO/KO littermates (n = 18). No differences are seen between genotypes. For all three charts, results are presented as means +/− s.e.m. Exact statistical values for each comparison are listed in Supplementary Table S1. (**c**) ABR thresholds in the same mice at the same frequencies, 1 DPN. Foxo3+/+ littermates (black, n = 17) have elevated thresholds at 12, 16, 24 and 32 kHz. Foxo3KO/KO littermates (pink, n = 18) have no detectable hearing at all five frequencies. S.e.m. bars were plotted for the Foxo3KO/KO data, but are too small to see. (**d**) ABR thresholds in the same mice at the same frequencies, 14 DPN. Foxo3+/+ littermates (black, n = 17) have largely recovered from noise exposure. Foxo3KO/KO littermates (pink, n = 18) have severe hearing loss and no significant recovery.

To determine if cochlear function is protected by FOXO3, we exposed 17 Foxo3+/+ FVB/n and 18 Foxo3KO/KO littermates for 30 minutes to an octave band of noise centered at 12 kHz and presented at 105 dB SPL. This noise dose is approximately 70% of the energy level used in published assays with CBA/CaJ mice^10^. This noise level does not substantially affect permanent auditory thresholds in FVB/nJ mice^36^. It also fails to eliminate high-frequency synapses^36^. All animals of both genotypes received three hearing tests (Fig. 1a) measuring five frequencies. It should be noted that throughout this paper, the analysis compares Foxo3+/+ to Foxo3KO/KO, and as such does not address haploinsufficiency. The 3-way ANOVA for all ABR measurements gave a p-value of 4 × 10^−11^ and an F value of 8.7. Post-hoc pairwise comparisons were performed using Bonferroni adjustments and are listed in Supplementary Table S1. Prior to noise exposure, Foxo3+/+ and Foxo3KO/KO littermates had identical hearing thresholds (Fig. 1b, Supplementary Table S1 and ref.^35^). One day post noise exposure (1 DPN), Foxo3+/+ littermates had significant auditory threshold shifts at 12, 16, 24, and 32 kHz, but not at 8 kHz (Fig. 1c, black, n = 17, Supplementary Table S1). In contrast, nearly all Foxo3KO/KO mice lacked detectable auditory thresholds at each frequency tested (Fig. 1c, pink, n = 18, Supplementary Table S1). Foxo3+/+ littermates had largely recovered their auditory thresholds by 14 DPN, although at 32 kHz they had incurred a slight permanent elevation on average (Fig. 1d, black, n = 17, Supplementary Table S1). No significant recovery was observed for their Foxo3KO/KO littermates at 14 DPN (Fig. 1d, pink, n = 18, Supplementary Table S1). At 14 DPN, all Foxo3KO/KO mice had significantly higher thresholds at all five frequencies when compared to their Foxo3+/+ littermates (Fig. 1d and Supplementary Table S1). These data strongly support a model where FOXO3 is required for auditory function after noise exposure.

### Hair cell survival, stereociliary morphology, and outer hair cell function after noise exposure in Foxo3KO/KO mice

**Figure 2:**
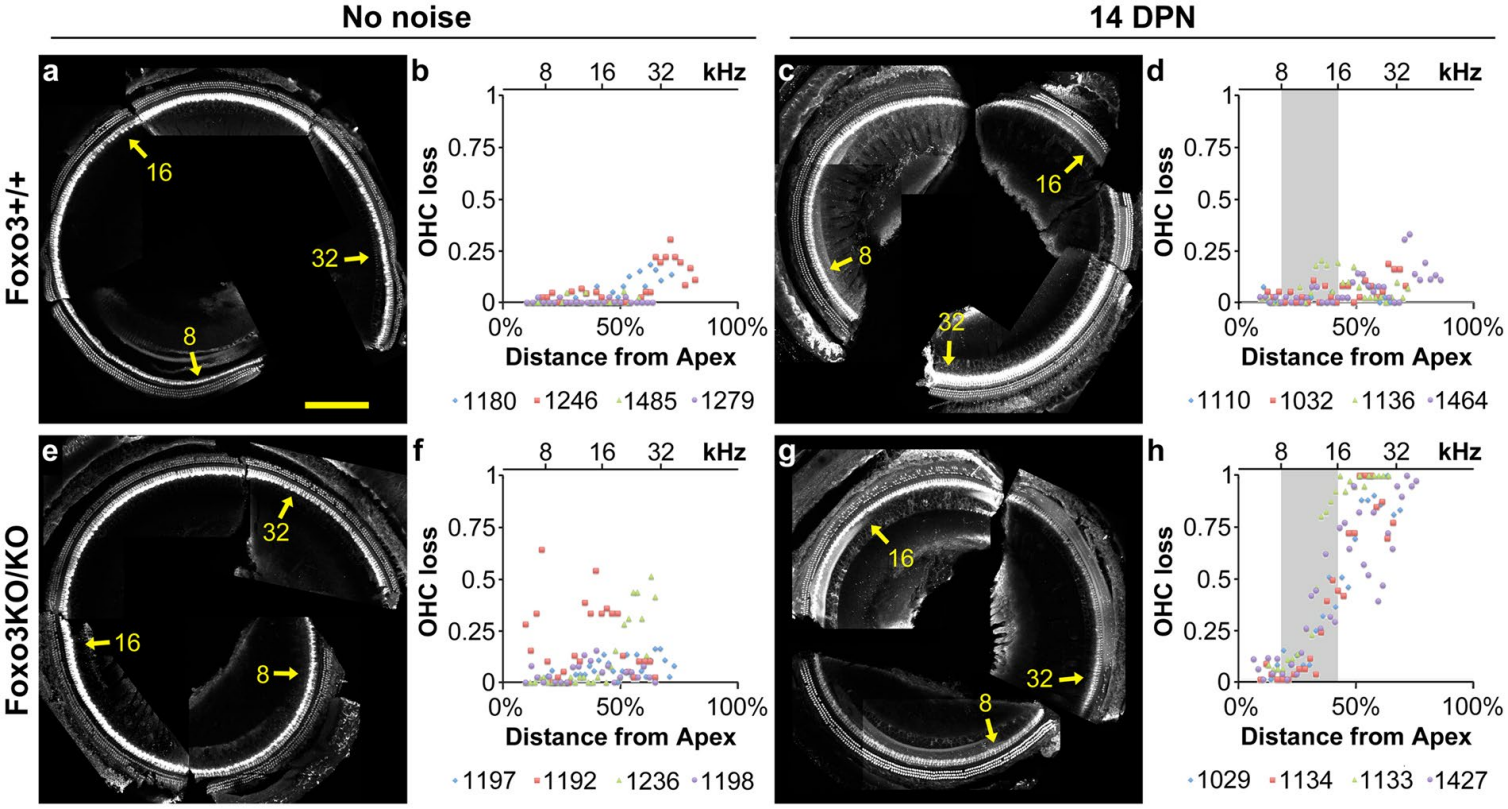
Outer hair cell loss is extensive in the Foxo3KO/KO after noise exposure. Inner and outer hair cells were visualized with antibodies to MYO7a and OCM in mapped, microdissected cochleae from Foxo3+/+ mice (**a**,**c**, white) and Foxo3KO/KO mice (**e**,**g**, white). The fraction of missing outer hair cells was determined for 100 micron segments along the mapped length and plotted in cochleograms (**b**,**d**,**f**,**h**, y-axis) against the percent distance from the cochlear apex (x-axis). The approximate frequencies are noted at the top of the graphs, and with yellow arrows on cochlear images. The legend for each cochleogram lists the number designation for each animal used (4 per condition and treatment); the first number listed is the representative display image. Animals that received auditory testing, but were not exposed to noise (**a**,**b**,**e**,**f**) are compared to animals that received both (**c**,**d**,**g**,**h**). The noise band (8–16 kHz) is shaded (**d**,**h**) for reference. Scale bar = 200 microns.

To identify potential causes of hearing loss after noise exposure, we quantified hair cell survival in Foxo3KO/KO mice at 14 DPN, comparing it to the no noise condition. Adult mice express FOXO3 in both inner and outer hair cells^35^. Cochleograms were used to assess survival of outer hair cell loss along the cochlear length. Foxo3+/+ littermates without noise exposure have a low basal level of outer hair cell loss, peaking at about 25% in the 32 kHz region (Fig. 2a,b). At 14 DPN, outer hair cell loss is unchanged in Foxo3+/+ littermates (Fig. 2c,d). Two of four Foxo3KO/KO mice without noise exposure have sporadic outer hair cell loss (Fig. 2e,f, note animals #1192 and #1236), and two of the four animals analyzed were fairly similar to Foxo3+/+ littermates (Fig. 2e,f; note animals #1197 and #1198). However, at 14 DPN, dramatic and specific outer hair cell losses were seen in all four animals, starting around the 16 kHz region and extending to the higher frequencies (Fig. 2g,h). No significant loss of inner hair cells was observed for either genotype or condition (Fig. 2a,c,e,g). These data indicate that FOXO3 is required for outer hair cell survival after noise exposure in a frequency-dependent fashion.

**Figure 3:**
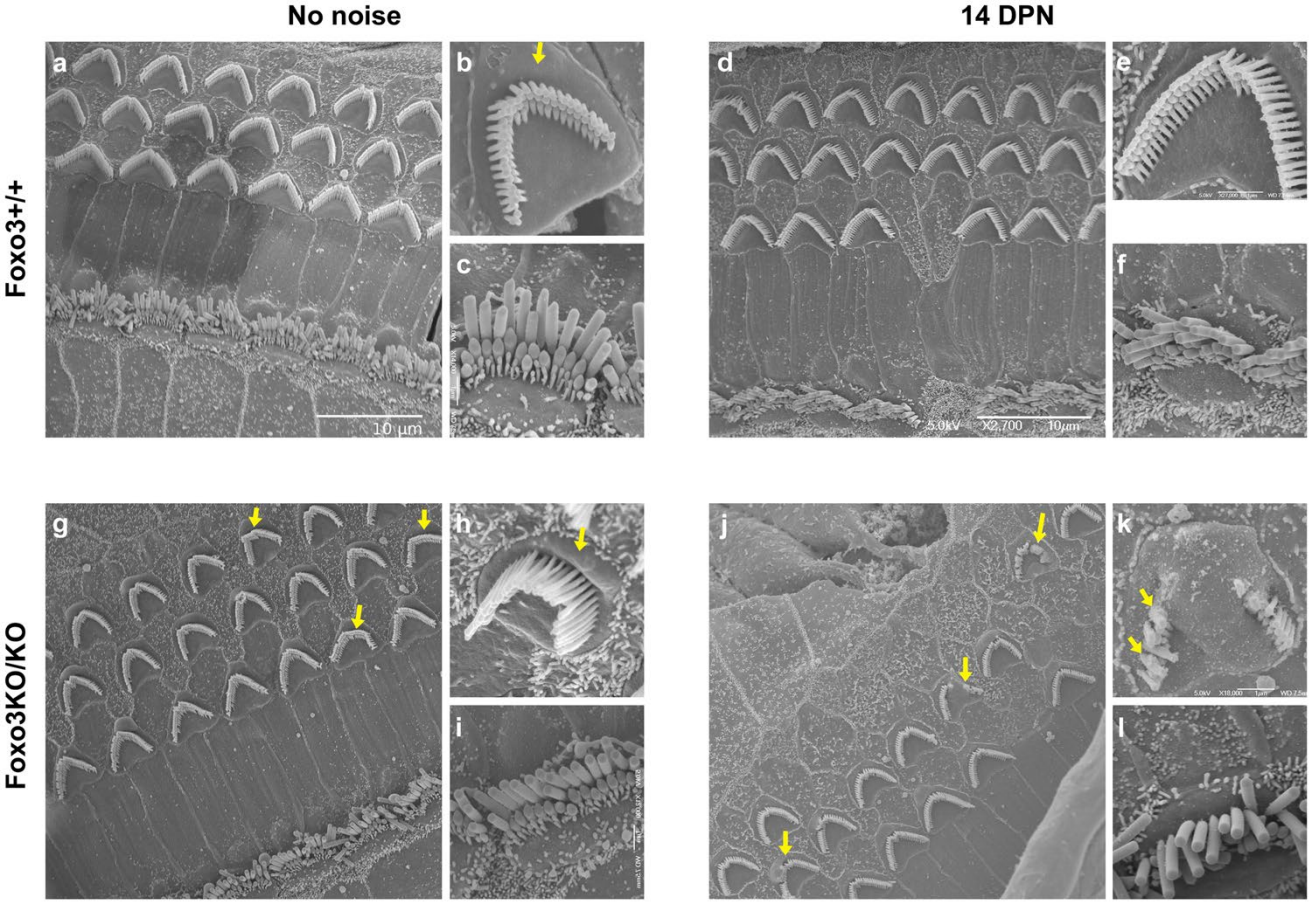
Morphology of inner and outer hair cell stereocilia. Stereociliary bundle morphology of outer (**b**,**e**,**h**,**k**) and inner hair cells (**c**,**f**,**i**,**l**) from control Foxo3+/+ mice (**a**–**c**) and Foxo3+/+ mice at 14 DPN (**d–f**) are compared to control Foxo3KO/KO littermates (**g**–**i**) and Foxo3KO/KO littermates at 14 DPN (**j**–**l**). SEM images are taken from the region corresponding to 12–16 kHz, i.e. within the noise band. In (**b**) and (**h)**, arrows indicate the apical cuticular region of outer hair cells. In (**g)** and (**j)**, arrows indicate outer hair cells with stereociliary dysmorphia. In (**k)**, arrows highlight fused stereocilia on a damaged, surviving outer hair cell.

As noise exposure is known to damage stereocilia (reviewed in ref.^37^), we investigated the morphology of inner and outer hair cell stereocilia in Foxo3+/+ and Foxo3KO/KO mice, both without noise exposure and at 14 DPN. Here we used scanning electron microscopy in the 12–16 kHz region, where outer hair cells persist in the noise-damaged Foxo3KO/KO. Foxo3+/+ littermates had well-formed stereociliary bundles in both conditions (Fig. 3a–f). Areas where outer hair cells were lost at 14 DPN had well-formed epithelial scars (Fig. 3d). Foxo3KO/KO stereociliary bundles were largely normal without noise exposure (Fig. 3h,i), but a few outer hair cells had oddly formed bundle V-shapes (Fig. 3g, arrows). Moreover, 33% of Foxo3KO/KO outer hair cells had prominent cuticular bulges on the apical side (n = 124 cells from 2 cochleae), whereas only 4% of Foxo3+/+ outer hair cells had similar bulges (Fig. 3, cf. b with h, arrows, n = 90 cells from 2 cochleae). At 14 DPN, Foxo3+/+ outer hair cells had normal looking stereocilia (Fig. 3d–f) and apical bulges were rare (7%, n = 70 cells from 2 cochleae). Many normal-looking stereocilia were also observed on Foxo3KO/KO outer hair cells in this region (Fig. 3j). However, a few Foxo3KO/KO outer hair cells displayed damaged bundles (Fig. 3j, arrows), with fused and stunted stereocilia (Fig. 3k, arrows). 61% of outer hair cells surveyed had apical bulges in these samples (Fig. 3j, lowest arrow, n = 66 cells from 2 cochleae). Inner hair cell bundles looked largely normal (Fig. 3l). These data suggest that prior to noise exposure, Foxo3KO/KO outer hair cells may experience some stress, resulting in cuticular bulges. Moreover, after noise exposure, a few surviving Foxo3KO/KO outer hair cells in the noise band have persistent structural injury.

**Figure 4:**
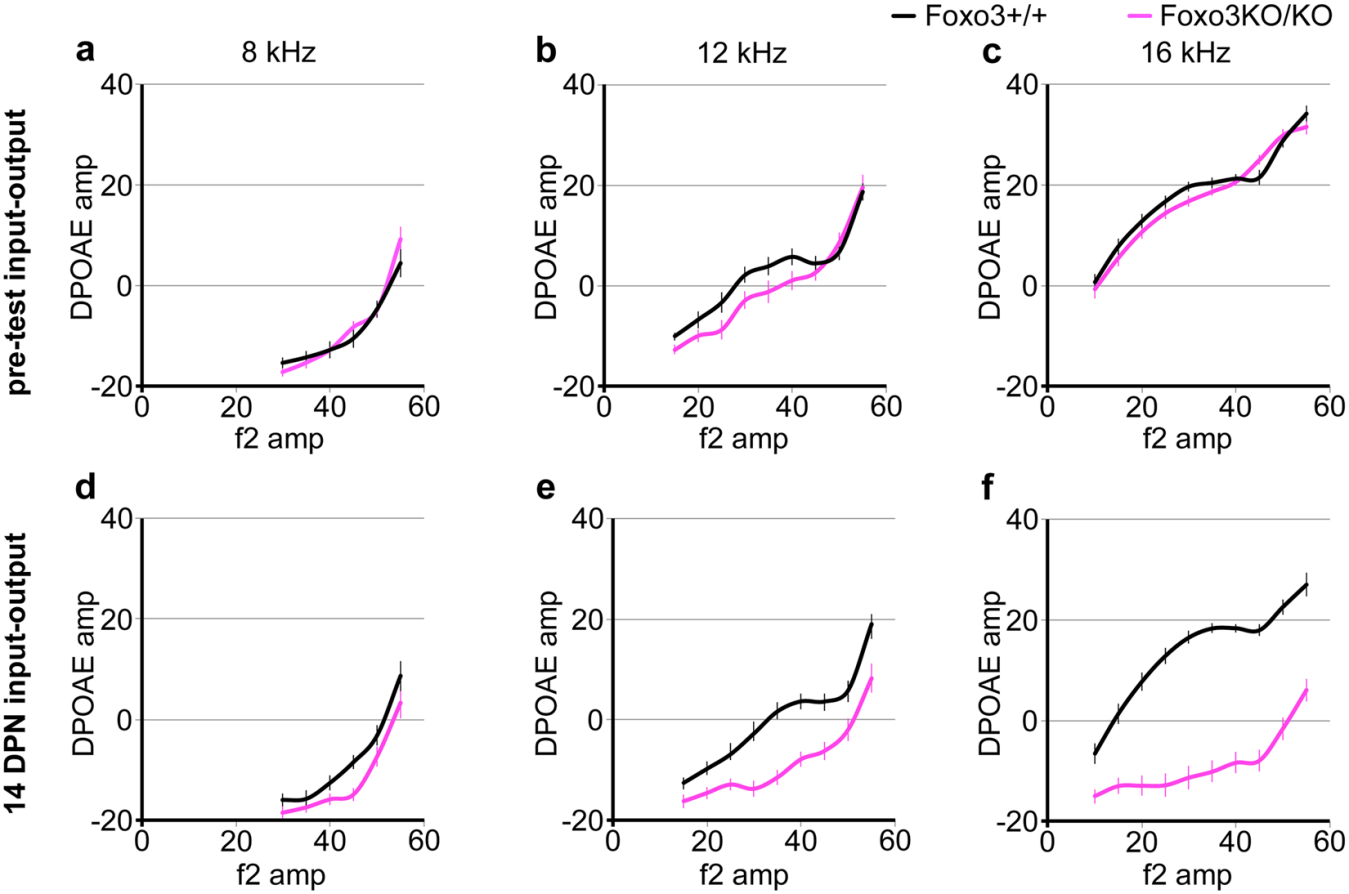
DPOAE input-output functions of regions with surviving outer hair cells. F2 amplitudes are plotted along the x-axis for 8 kHz (**a**,**d**), 12 kHz (**b**,**e**) and 16 kHz (**c**,**f**) for Foxo3+/+ littermates (black) and Foxo3KO/KO littermates (pink). DPOAE output amplitudes are plotted on the y-axis. Pre-test data are displayed (**a**–**c**), as are data from 14 DPN (**d**–**f**) from the same mice. Data are from 27 Foxo3+/+ mice and 30 Foxo3KO/KO mice. Each point is mean +/− s.e.m.

To assess the functionality of the surviving outer hair cells, we plotted the input-output functions for the distortion product otoacoustic emission (DPOAE) from 8, 12, and 16 kHz. Reduced DPOAE emissions at identical f2 amplitudes are a hallmark of lost or dysfunctional outer hair cells. Prior to noise exposure, we found that Foxo3KO/KO mice on average have slightly reduced input-output functions at 12 kHz (Fig. 4b, n = 30 Foxo3KO/KO and 27 Foxo3+/+), consistent with previous findings^35^ and the observed sporadic outer hair cell loss in unexposed Foxo3KO/KO mice (Fig. 2e,f). At 14 DPN, obvious reductions in DPOAE amplitude were observed at 12 and 16 kHz (Fig. 4e,f), but only minimal reductions were observed at 8 kHz (Fig. 4d). These data suggest that low frequency outer hair cells may retain function in the noise-exposed Foxo3KO/KO, even though no ABR thresholds are observed. Thus, while the loss of, and persistent damage to, mid- and high-frequency outer hair cells are striking findings in the Foxo3KO/KO after noise damage, they are not sufficient to explain the severe hearing loss phenotype.

### Synaptic characterization and spiral ganglion neuron survival after noise exposure in Foxo3KO/KO mice

**Figure 5:**
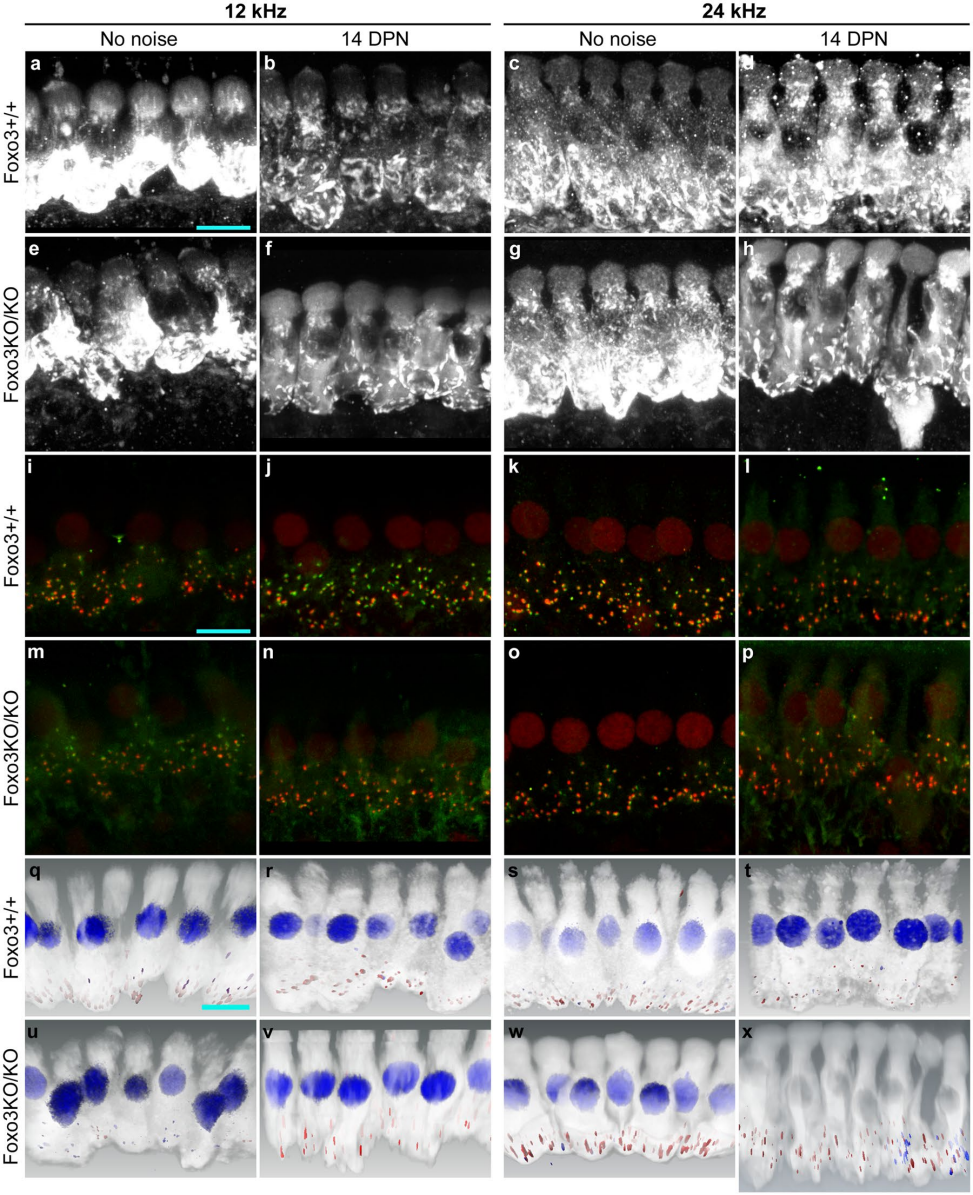
Analysis of Foxo3KO/KO inner hair cell morphology at 14 DPN. (**a**–**h**) Inner hair cells were visualized with anti-Myo7a (white) in confocal analysis. Conditions, genotypes, and frequency regions are as indicated: a, c, e, g show inner hair cells with no noise treatment, whereas b, d, f, h show inner hair cells at 14 DPN. Foxo3+/+ inner hair cells are depicted in a–d, and Foxo3KO/KO inner hair cells in (**e**–**h**). 12 kHz (**a**,**b**,**e**,**f**) and 24 kHz (**c**,**d**,**g**,**h**) are both depicted. Similar results were observed in 3–4 cochlear samples of each condition, genotype, and frequency. Cyan scale bar: 10 microns. (**i**–**p**) Same inner hair cells, with anti-CTBP2 (red) to reveal pre-synaptic ribbons and anti-GRIA2 (green) to reveal receptor patches. (**i**,**k**,**m**,**o**) Show inner hair cells with no noise treatment, whereas (**j**,**l**,**n**,**p**) show inner hair cells at 14 DPN. Foxo3+/+ inner hair cells are depicted in (**i**–**l**) and Foxo3KO/KO inner hair cells in m-p. 12 kHz (**i**,**j**,**m**,**n**) and 24 kHz (**k**,**l**,**o**,**p**) are both depicted. Cyan scale bar: 10 microns. (**q**–**x**) After Amira 3D reconstruction and analysis of the same confocal stacks, paired ribbon synapses, e.g. CTBP2+ structures associated with GRIA2+ structures (red) are distinguished from unpaired CTBP2+ structures (blue). MYO7 immunofluorescence is depicted in white. The organization of condition, genotype, and frequency regions is the same as (**a**–**h**) and (**i**–**p**). Cyan scale bar: 10 microns, approximately.

We examined inner hair cell morphology in the noise-exposed Foxo3KO/KO. Mapped, microdissected cochlear turns were stained with anti-MYO7a. Some background staining was observed, not atypical for this kind of whole mount preparation (Fig. 5a–p). Foxo3+/+ inner hair cells from both the 12 and 24 kHz turns displayed a characteristic gourd-like morphology with wide cuticular projections above the nucleus (Fig. 5a,c). MYO7a morphology was fairly similar at 14 DPN, compared to the no noise condition, in Foxo3+/+ littermates (Fig. 5a–d), although subtle changes in protein levels and cellular distribution may have occurred. Similar results were seen in a previous study^36^. Inner hair cells from Foxo3KO/KO littermates were similar in appearance to Foxo3+/+ inner hair cells at 12 and 24 kHz in the absence of noise treatment (Fig. 5e,g). At 14 DPN, occasional Foxo3KO/KO inner hair cells at 24 kHz had somewhat altered morphology, as revealed by MYO7a immunofluorescence (Fig. 5h). Their apical projections had thinned, and the basal portion of the inner hair cell appeared displaced (Fig. 5h). Notably, inner hair cell morphology at 12 kHz appeared relatively normal in the noise-exposed Foxo3KO/KO (Fig. 5f).

**Figure 6:**
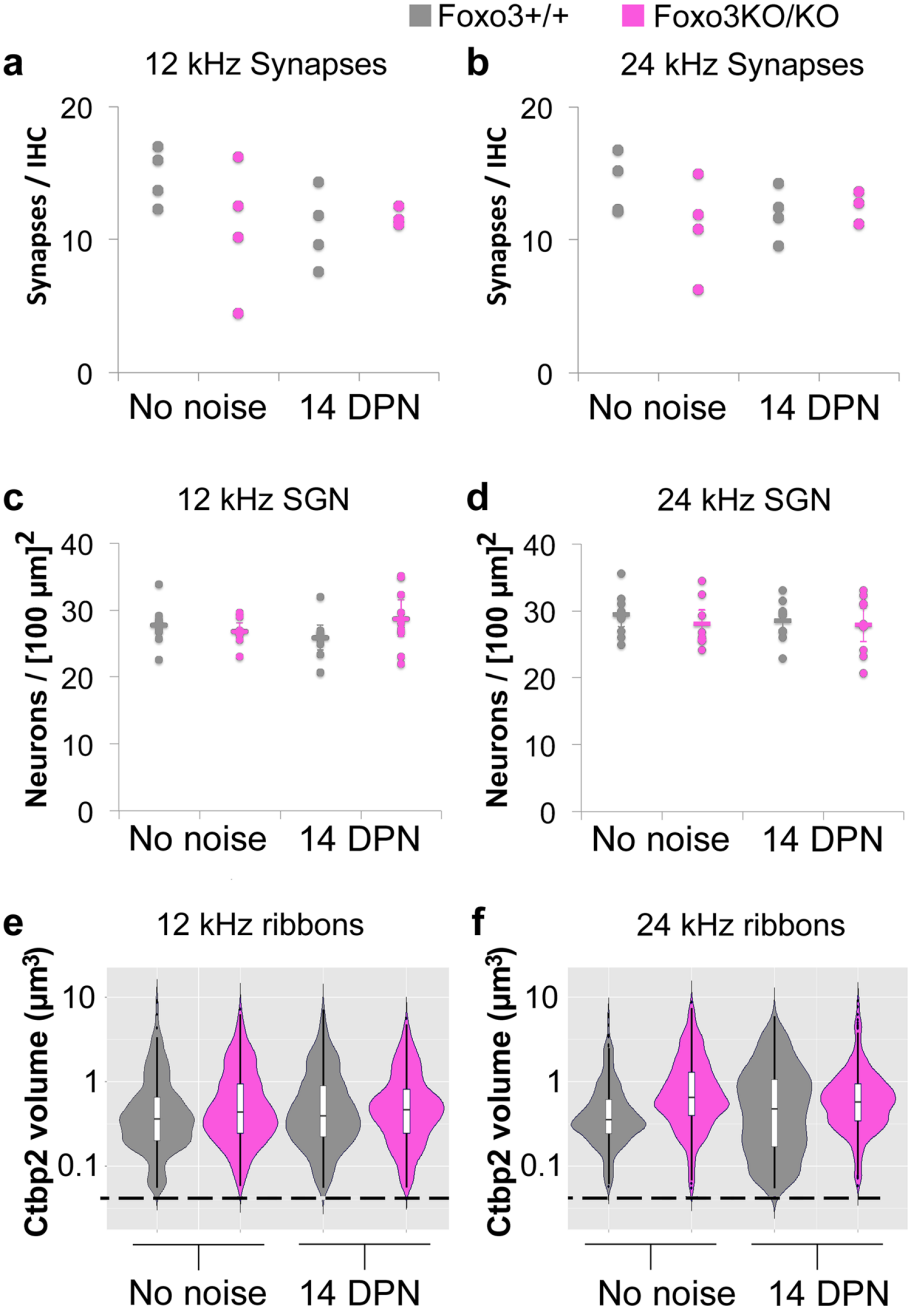
Quantification of synaptic numbers, neurons, and ribbon volumes. (**a**,**b**) Counts of algorithmically identified, paired synapses obtained from confocal stack 3D reconstructions of 12 kHz inner hair cells (**a**) are compared for Foxo3+/+ (gray) and Foxo3KO/KO (pink) animals without noise treatment or 14 DPN, as indicated. Similarly obtained counts for 24 kHz inner hair cells are depicted in (**b**). 3–4 organs were imaged for each condition and genotype; one dot is displayed for the average from 5–8 inner hair cells. (**c**,**d**) Spiral ganglion neuron density, relative to the area of Rosenthal’s canal in transverse sections, is shown for the 12 kHz (**c**) and 24 kHz (**d**) cochlear regions. 2–3 sections from each of 3 individual cochleae were quantified and are plotted as dots, with the mean represented as a line. No differences were seen between genotypes. (**e**,**f**) Distribution of volumes from all paired Ctbp2+ ribbon structures from 12 kHz (**e**) and 24 kHz (**f**) inner hair cells from animals without noise treatment (No noise) or 14 DPN, as indicated. Foxo3+/+ Foxo3+/+ (gray) ribbons are contrasted with Foxo3KO/KO (pink). The dotted line represents a filter imposed by Amira which excludes synaptic components smaller than 0.05 µm^3^. Statistical treatments are reported in the Results section.

Noise exposure drives auditory synaptic loss in animal models^10,36,38,39^. Our noise exposure, 70% of the energy used in those studies, is insufficient to eliminate synapses in either 12 or 24 kHz regions in control studies^36^. Thus, we were curious as to whether loss of FOXO3 function potentiated synaptic loss in this system. Anti-CTBP2 and anti-GRIA2 staining were used to reveal synaptic ribbons and post-synaptic receptor patches respectively (Fig. 5i–p). CTBP2 is also present in the nucleus of most inner hair cells. Synaptic pairs were observed in the noise-treated Foxo3KO/KO (Fig. 5n,p). These confocal data were imported into Amira for further analysis (Fig. 5q–x). Paired synapses were identified using the Amira XImagePAQ. Per hair cell, no difference was seen in numbers of paired synapses for either genotype, condition or frequency (Fig. 6a,b). Note that CTBP2 nuclear labeling in the noise-exposed Foxo3KO/KO inner hair cells at 24 kHz was reduced relative to synaptic labeling and thus does not appear in the reconstruction (Fig. 5x).

**Figure 7:**
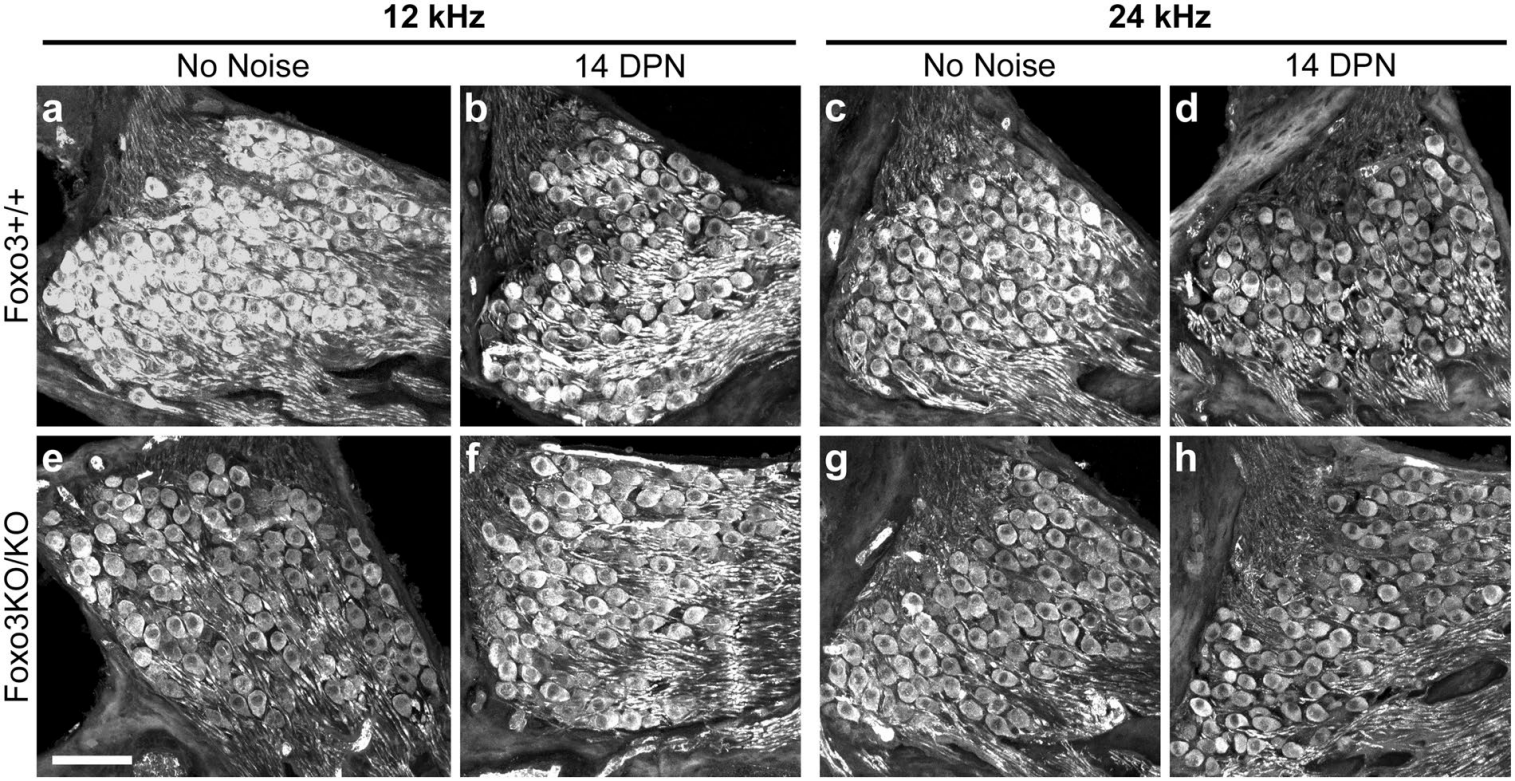
Spiral ganglion neuron survival is comparable between genotypes at 14 DPN. Cryosections of cochlear samples were oriented to reveal 12 kHz (**a**,**b**,**e**,**f**) and 24 kHz (**c**,**d**,**g**,**h**) spiral ganglia neurons, stained with anti-Tuj1+ (white), and imaged on a confocal microscope. Foxo3+/+ littermates (**a**–**d**) are compared to Foxo3KO/KO littermates (**e**–**h**). Animals that received no noise treatment (**a**,**c**,**e**,**g**) are compared to animals at 14 DPN (**b**,**d**,**f**,**h**). Scale bar = 100 µm.

Spiral ganglion neurons are required for hearing, and mouse spiral ganglion neurons express FOXO3^35^. We assessed spiral ganglion neuron survival in the Foxo3KO/KO mouse at 14 DPN, comparing results to Foxo3+/+ spiral ganglion neuron survival at 14 DPN as well as both genotypes without noise exposure. Spiral ganglion neurons were quantified on cross-sections of Foxo3+/+ and Foxo3KO/KO cochleae oriented to reveal the 12 and 24 kHz turns (Fig. 7). No loss of spiral ganglion neurons was observed in the Foxo3KO/KO under either condition (Fig. 6c,d). These data suggest that FOXO3 may be dispensable for spiral ganglion neuron survival after noise exposure. Other possibilities will be addressed in the Discussion section.

Previously, we found that after noise exposure, preserved auditory synapses may modulate their ribbon sizes^36^. Although no modulation was previously observed at 12 kHz, the average ribbon size increased moderately at 24 kHz^36^. Such variations can be displayed with logarithmic violin plots, where the shape of the “violin” depicts the kernel density estimation (smoothed histogram of probabilities) for the population. When the distributions of ribbon volumes for Foxo3+/+ and Foxo3KO/KO inner hair cells were plotted for 12 kHz, they were similar, irrespective of noise treatment (p = 0.10, ANOVA, Fig. 6e). For each genotype and treatment, the most prevalent size was around 0.3–0.5 µm^3^ (Fig. 6e, widest point of each “violin”). At 24 kHz, significant differences were observed in the ribbon size distribution (p = 1.4 × 10^−6^, ANOVA). The most prevalent size of unexposed Foxo3+/+ ribbons was also 0.3 µm^3^ (Fig. 6f, left gray “violin”). Noise exposed Foxo3+/+ ribbons, in contrast, are on average larger, with a new most prevalent size at 1.0 µm^3^ (p = 0.029, Wilcoxon with continuity correction, Fig. 6f, widest point of right gray “violin”), indicating a modulation in ribbon size. At 24 kHz, the most prevalent size of unexposed Foxo3KO/KO ribbons was around 0.5 µm^3^, which was significantly different from unexposed Foxo3+/+ (p = 2 × 10^−16^, Wilcoxon with continuity correction). Modulation in ribbon size was seen in the Foxo3KO/KO after noise exposure (Fig. 6f, cf. pink “violins,” p = 0.012, Wilcoxon with continuity correction). Although the significance of the ribbon size modulation is not known, it is an adaption by Foxo3+/+ inner hair cells after noise exposure that also occurs in Foxo3KO/KO inner hair cells.

### Foxo3 immunoreactivity in cochlear sections from human cadaver specimens

**Figure 8:**
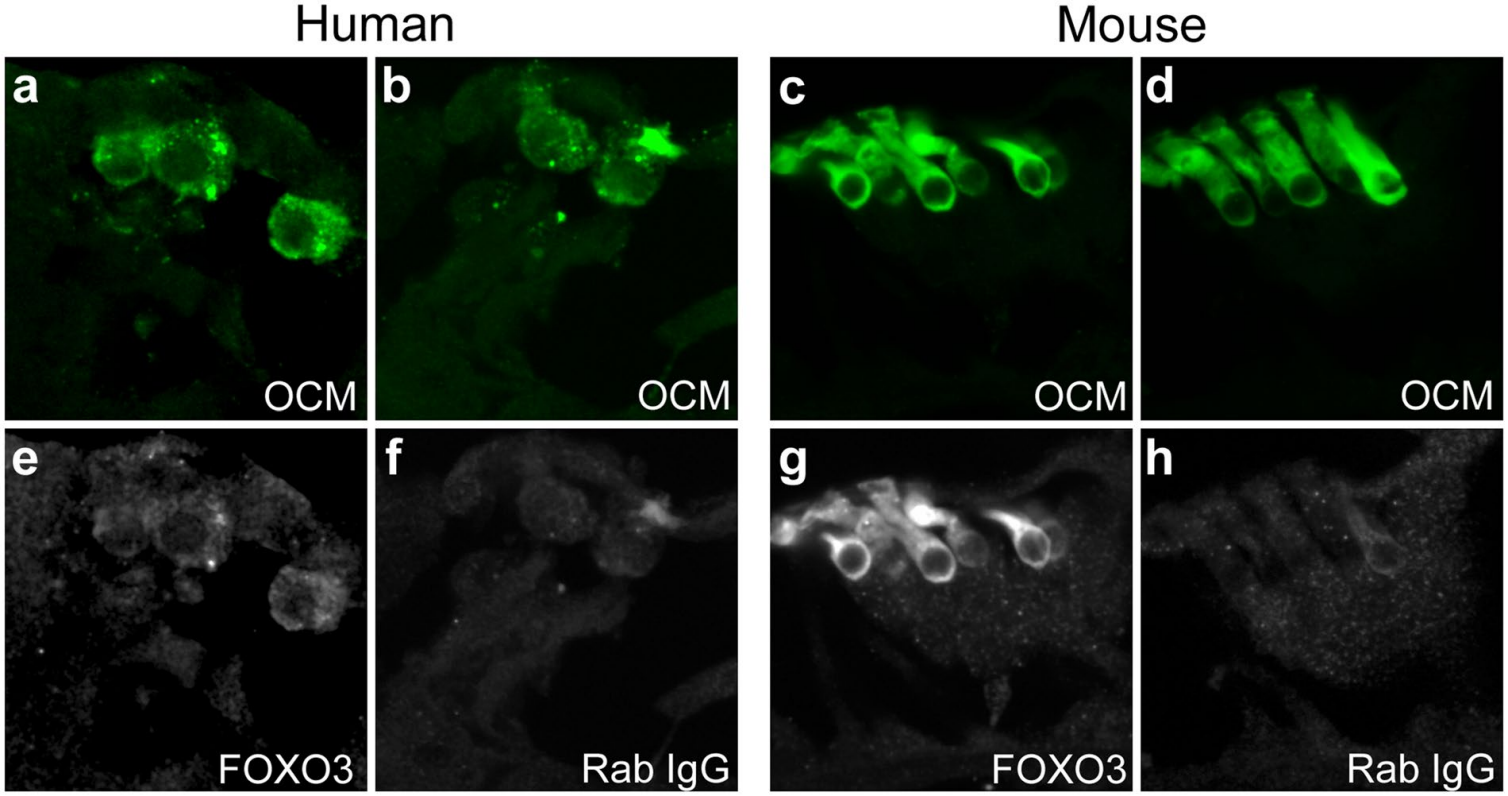
Foxo3 immunofluorescence in the adult human cochlea. Adult human (**a**,**b**,**e**,**f**) and mouse (**c**,**d**,**g**,**h**) sections, both obtained from the Otopathology Laboratory, were probed with antibodies against OCM to reveal outer hair cells (**a**–**d**, green). A validated anti-FOXO3 antibody (**e**,**g**, white) was used to reveal FOXO3 protein, in comparison to a normal rabbit IgG control (**f**,**h**, white).

FOXO3 is implicated in the regulation of human longevity through many studies of extremely old individuals^20,40–44^. Foxo3 mRNA was reported in the Morton human fetal cochlea cDNA library^45^. However, its cellular location in the human cochlea has yet to be established. Sections of human cochleae, preserved at 3 hours post-mortem, were stained with an antibody against OCM and a commercially validated antibody for FOXO3, and compared to serial sections stained with anti-OCM and control rabbit IgG (Fig. 8a,b,e,f). Low levels of FOXO3 immunofluorescence were observed in the human outer hair cells, in the image matched to the negative control (Fig. 8e, cf. 8f). Only cytoplasmic staining was observed (Fig. 8e). Similar results were obtained from mouse sections prepared at the same time (Fig. 8c,d,g,h). Cytoplasmic localization of FOXO3 was previously reported in cochlear cells^35^. These data suggest that FOXO3 protein is present in sensory cells of the human cochlea, supporting the contention that it may also play a role in human hearing.

## Discussion

We show that the transcription factor FOXO3 is required for auditory function in all frequencies tested after noise exposure in mice. This hearing loss correlates with widespread losses of mid- and high-frequency outer hair cells. Moreover, persistent structural damage is observed in a few of the remaining outer hair cells within the noise band. Inner hair cells, spiral ganglion neurons and auditory synapses are retained in full number at 14 DPN. Taken together, these data indicate that FOXO3 is required for hearing after noise exposure and specifically implicate FOXO3-driven transcription targets in outer hair cell survival. This is significant, in that we also report FOXO3 immunofluorescence in human outer hair cells. However, the presence of DPOAE function at 8 kHz, concurrent with an absence of ABR at the same frequency, suggest that additional, unknown defects are present in the noise-exposed Foxo3KO/KO.

The identification of genes impacting late-onset hearing loss in humans has proven challenging^46^, even though hearing loss is far more common in the elderly^1^. Human GWAS has implicated only a handful of genes, such as the metabolic glutamate receptor GRM7, in late-onset hearing loss^46–50^. Some mutations in the mitochondrial genome confer a susceptibility to hearing damage from aminoglycosides (reviewed in ref.^51^). Notably, allelic variations in SOD2, CAT, and PON2 have been associated with hearing loss from noise in industrial workers in candidate gene studies^52–54^. Genetic studies of late-onset hearing loss in humans is complicated by the interplay between human genetic diversity and the many decades of experience that characterize the human lifespan. Noise exposure experiments using congenic mouse lines controls for genetic diversity, enables reproducible damage models, and allows for genetic analysis of hearing recovery after noise.

We find that outer hair cell survival is compromised in the Foxo3KO/KO after noise exposure. Others have reported on mouse mutations that significantly impact outer hair cell survival after noise. A recent report on mouse *pejvakin* mutants (*Pjvk*-KO) revealed that their enhanced damage after noise could be attributed to a failure in the regulation of peroxisome number in outer hair cells^15^, contributing to our understanding of the gene deficit responsible for DFNB59^14^. In another case, a point mutation in *Cdh23* ^55^ confers sensitivity to noise exposure in several strains of mice^56^. Complete loss of function in *CDH23* causes congenital hearing impairment in USH1D syndrome^57,58^. These important findings shed light on the function of genes already implicated in early-onset hearing loss. In other systems, FOXO3 has a fundamental role in promoting the oxidative stress response^25,28^, one possible mechanism for the effects we report here. Thus, our findings with FOXO3 expand the set of proteins that are implicated in cochlear homeostasis.

Importantly, low frequency outer hair cells (8 kHz) are apparently not impacted by noise exposure in the Foxo3KO/KO: they both survive (Fig. 2) and retain function (Fig. 4). Others have shown that low frequency outer hair cells are more resistant to stress from noise or ototoxic drugs, compared to more susceptible high frequency outer hair cells^59–64^. This may be interpreted for the presence of an unknown “buffering” capacity for stress present in the apex of the cochlea. Our results would indicate that this “buffering” capacity is independent of FOXO3.

Prior to noise exposure, Foxo3KO/KO outer hair cells have detectable abnormalities. This is consistent with the depressed input-output functions previously reported^35^. Two of four Foxo3KO/KO control animals displayed sporadic outer hair cell losses in cochleogram analysis (Fig. 2). In the analysis of the *pejvakin* mutants, greater outer hair cell losses were observed in pups from larger litters^15^. In our SEM analysis, 33% of Foxo3KO/KO outer hair cells had bulging cuticular plates without noise exposure (Fig. 3). Such bulges are reminiscent of those found in early studies of noise damage in the chinchilla^61^. We speculate that the ambient noise level within the home cage could sometimes be sufficient to affect outer hair cell morphology and survival in the absence of FOXO3.

We report that in the noise-exposed Foxo3KO/KO, fused and malformed stereocilia occasionally persist on surviving outer hair cells in the noise band (Fig. 3). Fused stereocilia were also reported in early studies of noise damage^61^. We note that the damaged outer hair cells phenocopy a conditional loss-of-function *Cdc42* mutant^65^. Cdc42 is a member of the Rho sub-family of small GTPases that regulates the assembly of actin-rich cellular structures^66^. Of course, reduced actin assembly could be a consequence of problems in any of several cellular process, including energy metabolism, transport, and protein catabolism. However, a recent report showed that Foxo3KO/KO erythroblasts have aberrant expression of a gene cluster of cytoskeletal organization and cell polarization proteins^67^. We speculate that recovery from noise exposure may require new synthesis of these important proteins, and their dysregulation could affect Myo7a distribution.

Surprisingly, we find no substantial effect of the loss of FOXO3 on the survival of inner hair cells and spiral ganglion neurons, or on the retention of high-frequency inner hair cell synapses (Figs 5–7). Moreover, both Foxo3KO/KO and Foxo3+/+ inner hair cells in the 24 kHz region modulate their ribbon sizes after noise exposure (Fig. 6). There are several possible explanations for these negative findings. First, other genetic networks, such as the mitochondrial deacetylase SIRT3, might protect synapses from noise damage^16^. Second, we did not extend our observations to later time points, where spiral ganglion neuron losses become more evident^10^. Third, acute outer hair cell injury in the Foxo3KO/KO during noise exposure may result in reduced signaling to inner hair cells and spiral ganglion neurons, mitigating downstream damage. A final possibility harkens back to FOXO3’s best-defined role in neurons: perhaps FOXO3 is required for apoptosis in inner hair cells and spiral ganglion neurons. Conditional knockout experiments will be necessary to distinguish between these possibilities.

Although we were unable to identify the mechanism, we infer that the loss of FOXO3 has functional consequences for inner hair cells and spiral ganglion neurons for two reasons. First, the aging Foxo3KO/KO has elevated ABR hearing thresholds in the presence of DPOAE production^35^. Second, we report here that there is little ABR response at 8 kHz in the noise exposed Foxo3KO/KO (Fig. 1), even though we observe outer hair cells at this frequency (Figs 2 and 3) and detect DPOAE at 8 kHz (Fig. 4). Thus, the severe hearing phenotype in the noise-exposed Foxo3KO/KO cannot be explained by the loss of outer hair cells alone.

Identifying genetic factors that influence recovery from noise exposure is an important step forward in understanding NIHL. We propose that FOXO3 is such a candidate. Interestingly, caloric, oxidative or mechanical stress promote FOXO3 nuclear localization, whereas insulin signaling can promote FOXO3 cytoplasmic localization (reviewed in ref.^68^). We observed cytoplasmic FOXO3 localization in preparations of both human and mouse outer hair cells presented here (Fig. 8). Previously, we had found that FOXO3 translocates into the nucleus of cochlear cells after noise exposure^35^. Thus, FOXO3 may link cochlear activity to the expression of genes implicated in cochlear homeostasis, such as its well known targets in oxidative stress mitigation, SOD2 and CAT^28^. Of course, the presence of FOXO3 immunofluorescence in outer hair cells in human cochlear sections suggests these results may be pertinent to human noise induced hearing loss. Future work will focus on FOXO3 targets in the cochlea, with the goal of identifying gene networks that regulate homeostasis.

In conclusion, we show that the transcription factor FOXO3 is required for auditory function after noise exposure in adult mice in all frequencies tested. Foxo3KO/KO mice have extensive mid- and high-frequency outer hair cell loss after noise exposure; however, low frequency outer hair cells survive and appear to function. Inner hair cells, spiral ganglion neurons, and primary auditory synapses are all present in the Foxo3KO/KO after noise exposure. The presence of functional low frequency outer hair cells concomitant with the absence of auditory function at the same frequency indicate that an additional, unknown mechanism affects hearing in the Foxo3KO/KO after noise exposure. In neural damage paradigms such as stroke, FOXO3 promotes delayed apoptosis after stress. Thus, these findings show an unusual role for FOXO3 in hearing.

## Materials and Methods

### Animal usage

All experiments were performed in compliance with the US Department of Health and Human Services, and were approved by the University Committee on Animal Resources at the University of Rochester Medical Center. Heterozygous/homozygous FoxO3 (FoxO3+/2) mice (FVB;129S6-Foxo3tm1.1Rdp; RRID:MGI:2668335) were obtained from the Mutant Mouse Regional Resource Center, at the University of California at Davis (Davis, CA; stock no. 016132-UCD)^69^. Originally made on 129 Sv and bred three times to FVB/n, we bred the line a fourth time to FVB/nJ (RRID:IMSR_JAX:001800) to obtain heterozygote mutant mice. Heterozygotes with different parents were bred together to obtain both knockout and Foxo3+/+ littermates. Thirty-six *Foxo3KO/KO* and thirty-five Foxo3+/+ littermates, both male and female, were used in this study. Mice were given ample nesting materials and small houses within their home cage. Mice were assigned to conditions one of two ways: either an entire litter was designated for noise exposure or control conditions, or littermates were randomly assigned to conditions using a deck of cards. Same-sex littermates receiving the same treatments were housed together in twos and threes.

For genotyping, DNA was obtained from 2-mm tail samples that were digested overnight in Proteinase K (IBI Sciences) solution at 65 °C followed by phenol/chloroform extraction. IProof Taq (BioRad) was used in conjunction with a published protocol and primer sequences^70^.

### Noise exposure

Awake two month old mice were exposed to noise limited to the 8–16 kHz octave band at 105 decibels for 30 minutes. Mice were each placed into individual triangular wire mesh cages, 12 cm × 5 cm × 5 cm, in an asymmetric plywood box with a JBL2250HJ compression speaker and JBL2382A biradial horn mounted on the top. This apparatus was contained within a sound booth. The speaker was driven by a TDT RX6 multifunction processor and dedicated attenuator, and controlled with TDT RPvdsEx sound processing software. The sound level was calibrated with a Quest sound meter, Model 1900. Mice were exposed between the hours of 9 AM and 1 PM to control for circadian rhythm effects^71^, and the sound level was checked with an iPhone using the FaberAcoustical SoundMeter app and the iPhone’s internal microphone each morning before use. The iPhone was previously calibrated with the SoundMeter app software and a solid state 94 dB source (provided with the Quest sound meter) in a sound booth. Within the sound exposure box, we had marked three specific locations in the back of the box where the sound levels were highly consistent for the footprint of the mouse cage (+/− <0.5 dB) and always placed the mouse cages at these three locations. Sound levels were measured in the center location. Other areas of the box tended to be louder. Animals that moved their cages from the starting position were discarded.

### Auditory testing

Mice were tested at 7 weeks of age (pre-test) and again at P74; mice receiving noise exposure at P60 were also tested the next day to determine temporary threshold shifts (Fig. 1a). Auditory testing was conducted using a Smart EP Universal Smart Box (Intelligent Hearing Systems). Mice were anesthetized with an intraperitoneal injection of ketamine (80 mg/kg) in a sterile acepromazine/saline mixture (3 mg/kg). A 10B+ (high frequency transducer/stimulator) probe was placed at the opening to the external auditory meatus.

Auditory brainstem response (ABR) stimuli were 5-ms clicks, or 5-ms tone pips presented at 5 frequencies between 8 and 32 kHz. Stimuli began at 75 dB amplitude and decreased by 5 dB steps to 15–25 dB. 512 sweeps were averaged for each frequency and amplitude. Electrical responses were measured with three subdermal needle electrodes (Grass): one inserted beneath each pinna, and a third, the ground electrode, placed at the vertex. ABR thresholds for a particular frequency were determined by any part of the last visible trace (dB). The person scoring the waveforms was blinded to genotype, condition and time point.

For distortion product otoacoustic emissions (DPOAE), we measured the amplitude of evoked otoacoustic emissions to paired pure tones of frequencies f1 and f2, where f1/f2 = 1.2 and the f1 level was 10 dB above f2. Thirty-two sweeps were made in 5 dB steps starting with f1 at 20 dB and ending at 65 dB.

### Tissue preparation for immunostaining

Cochlear organs were dissected out of freshly euthanized animals. Their stapes were removed, and a hole was made in their apical tips to allow for adequate fluid exchange. Tissues were immersed in 4% paraformaldehyde in PBS for at least overnight. Tissues were decalcified in 0.1 M EDTA at 4 °C on a rotating platform for four days. For cryosectioning, tissues were immersed in 30% sucrose in PBS overnight, embedded in OCT, and frozen in liquid nitrogen. For neuron quantification, the cochlea was oriented to present the 12 and 24 kHz regions in transverse section as it was embedded, and cryosectioned at 20 microns. For hair cell counts and synapse analysis, whole mount preparations were microdissected into turns as previously described^72^, mapped using the ImageJ plug-in from Massachusetts Eye and Ear Infirmary, and immunostained for analysis.

Sections of human temporal bone were kindly donated by Dr. Joseph Nadol from the Otopathology Lab at Massachusetts Eye and Ear Infirmary of Harvard Medical School. Temporal bones were processed for celloidin sectioning as previously described^73^. Sections were from two human individuals. The first was a 70 year old male, with no information on hearing status, whose tissue was preserved 3 hours post-mortem. The second was a 58 year old male, who had one audiogram done 11.5 years before death that was within normal range, whose tissues were preserved 28 hours post-mortem. These sections, as well as sections from two control mice, were boiled in citric acid for 15 minutes before blocking for one hour in 5% donkey serum in TBST at room temperature. Primary and secondary antibody incubations were both performed overnight at 4 °C in 5% donkey serum in TBST. Control rabbit IgG at the same concentration was used as a negative control for staining. Foxo3 was detected in the long red (647) channel to minimize tissue autofluorescence artifacts. Results are shown from the human specimen that was preserved three hours post-mortem. The second individual’s sample did not show any FOXO3 immunofluorescence (not shown), and morphology of the outer hair cells was poor (not shown).

### Antibodies

The following primary antibodies were used: mouse anti-Tubulin beta III isoform C-terminus, clone TUJ1 (1:500; Millipore; RRID:AB_2210524), goat anti-Oncomodulin (Ocm) antibody (1:1000; Santa Cruz; RRID:AB_2267583), rabbit anti-Foxo3 (1:1000; Thermo Scientific; RRID:AB_2544621), rabbit anti-Myosin7a (Myo7a) (1:200; Proteus; RRID:AB_10013626) mouse anti-Ctbp2 (aka C-Terminal Binding Protein 2; 1:200; BD Transduction Laboratories; RRID:AB_399431), and mouse anti-Gria2 (aka GluR2/GluA2; 1:2000; Millipore; RRID:AB_2113875). The following secondary antibodies, all purchased from Jackson ImmunoResearch, were used: Donkey Anti-Mouse 488 (1:500; RRID:AB_2340849), Donkey Anti-Rabbit 594 (1:500; RRID:AB_2340622), Donkey Anti-Rabbit 647 (1:200; RRID:AB_2340625), Donkey Anti-Goat 647 (1:200; RRID:AB_2340438), Alexa 594 Goat Anti-Mouse (IgG1, 1:500; RRID:AB_2338885), Alexa 488 Goat Anti-Mouse (IgG2a, 1:500; RRID:AB_2338855).

### Immunostaining

For anti-TuJ1 staining on mouse sections, blocking was carried out at room temperature for two hours in 0.5% Tween/5% donkey serum (Jackson) in Tris-buffered saline. Antibody incubations were carried out overnight at 4 °C in block solution. For whole mount staining, dissected mapped turns were immersed in 30% sucrose, flash frozen in liquid nitrogen, allowed to thaw, washed in room temperature Dulbecco’s PBS (Gibco), and blocked for one hour in 1% Triton/5% donkey serum in PBS. Primary antibody incubations of anti-Myo7a, anti-Ocm, anti-Ctbp2, and anti-Gria2 were performed at 37 °C for 20 hours. The tissue was washed in PBS, and secondary antibody incubation was performed at 37 °C for an additional 2 hours, with both Myo7a and Ocm labeled with 647-conjugated antibodies. This whole mount protocol was kindly provided by Leslie Liberman. All tissue was mounted using ProLong Gold (Fisher). Whole mounts were placed between two 50 mm coverslips for imaging.

### Confocal microscopy and image processing for figures

All imaging was done on an Olympus FV1000 laser scanning confocal microscope. ImageJ 64 (NIH) was used to Z-project maximal brightness in confocal stacks. ImageJ 64 was also used to quantify the area of Rosenthal’s canal in transverse images of the spiral ganglia. Photoshop (Adobe) was used to set maximal and background levels of projections for the construction of figures. Composite images for cochleograms were assembled in Photoshop by pasting each optical section into its own layer, and merging the pieces of the optical sections where hair cells were evident. Alternatively, projections of confocal stacks were used when individual inner hair cells could be clearly distinguished. Composite or projected images for the mapped regions of each organ were assembled in a single file in Photoshop. 100 micron lengths were pasted onto the images on the row of pillar cells, and outer hair cells were counted to determine the percent lost. Where the loss of outer hair cells was great enough (>30%), an average outer hair cell count, determined from low-frequency regions of the same cochlea, was used as the denominator.

### 3-D reconstructions of synaptic components and statistical analyses

To quantitatively assess synaptic number and distribution in inner hair cells, we used immunofluorescence and confocal microscopy in conjunction with three-dimensional modeling. Amira modeling was used to identify paired synapses for assessment of size and distribution. Anti-Ctbp2 labeled inner hair cell nuclei and pre-synaptic ribbon structures^74^, and anti-Gria2 was specific for the post-synaptic AMPA receptor^75^. Anti-Myo7a labeled inner hair cells^76^. We imported the confocal data Amira (Visualization Science Group) to generate 3-D reconstructions.

Ctbp2+, Gria2+and Myo7a+ staining volumes and their positions within the confocal stacks were identified computationally from Amira output files. Confocal data files (.oib) of Gria2+ (green), Ctbp2+ (red), and Myo7a+ (blue) stained inner hair cells, imaged at 200X on the FV1000, were opened in Amira. The inner hair cell region was cropped. A Myo7a isosurface was created from the data, appropriately thresholded, and exported. The Ctbp2 and Gria2 datasets were processed similarly, except that the isosurface was generated from the connected components region function. Methods using Amira were taught to us by the Liberman lab. All exported volumes were checked to ensure that volumes were within instrument resolution limits following threshold adjustments. Blind deconvolution of optical stack data was performed using the “maximum likelihood estimation” package for Amira. Briefly, point spread functions used for deconvolution were calculated using the optical parameters of the microscope (N.A. = 1.40, η = 1.516, UPlanSAPO 100x oil objective, 400–800 nm) and the wavelength of interest. Data were collected such that no saturated pixels were observed in fluorescence fields and z-axis spacing was in accordance with appropriate Nyquist sampling. The distinction for confocal deconvolution was utilized for all calculations and underwent no more than 10 iterations prior to convergence. To identify pairs of synaptic components, we used the new Amira XImagePAQ functionality, which can identify adjacent staining elements and output appropriate lists.

All statistical tests were performed in R64 using standard functions.

### Scanning electron microscopy

Cochleae were rapidly dissected out of the cranial bone one animal at a time to minimize the amount of time between death and fixation (typically 90 seconds) at room temperature. 400 µl of fixative, containing 4% paraformaldehyde and 2% gluteraldehyde in 0.1 M sodium cacodylate buffer, was gently pipetted through the open oval window, exiting through the hole made in the apical tip. Tissues were post-fixed at 4 °C on a rotating platform overnight, rinsed three times with distilled water, decalcified in 10% EDTA in 100 mM Tris pH 7.4 for three days, and then rinsed again. Cochlea turns were dissected, post-fixed in 1% osmium tetroxide for 2 hours at room temperature, and then processed through the thiocarbohydrazide-osmium tetroxide repeated procedure^77^. Samples were then dehydrated, critical point dried, mounted on support stubs with silver paint, and sputter coated with platinum. Imaging was carried out on a JEOL JSM6700F scanning electron microscope, operating at 5 kV.

## Electronic supplementary material

Supplementary Table S1
