# Supplementary material for: Severe hearing loss and outer hair cell death in homozygous Foxo3 knockout mice after moderate noise exposure
Source: Sci Rep. 2017 Apr 21;7:1054. doi: 10.1038/s41598-017-01142-3 (PMC5430619; doi:10.1038/s41598-017-01142-3)
Supplement: Supplementary file 1 — Supplementary Table S1 [file 41598_2017_1142_MOESM1_ESM.zip › Supplementary Table 1.doc]

**Supplementary Table S1.**

|  | **ABR thresholds Calculated P-values** | | | | |
| --- | --- | --- | --- | --- | --- |
|  | 8 kHz | 12 kHz | 16 kHz | 24 kHz | 32 kHz |
| WT  pre-test vs. 1 DPN | 0.11 | 6 x 10^-6^ | 7 x 10^-7^ | 1 x 10^-8^ | 1 x 10^-13^ |
| WT  pre-test vs. 14 DPN | 0.92 | 1 | 1 | 1 | 0.008 |
| KO  pre-test vs.1 DPN | <2 x 10^-16^ | <2 x 10^-16^ | <2 x 10^-16^ | <2 x 10^-16^ | <2 x 10^-16^ |
| KO  pre-test vs. 14 DPN | 2 x 10^-16^ | <2 x 10^-16^ | <2 x 10^-16^ | <2 x 10^-16^ | <2 x 10^-16^ |
| pre-test  WT vs. KO | 0.77 | 0.85 | 0.31 | 0.31 | 0.15 |
| 1 DPN  WT vs. KO | 2 x 10^-10^ | <2 x 10^-16^ | 4 x 10^-7^ | 9 x 10^-12^ | 0.15 |
| 14 DPN  WT vs. KO | 1 x 10^-9^ | <2 x 10^-16^ | 1 x 10^-15^ | <2 x 10^-16^ | 4 x 10^-10^ |

**Supplementary Table S1. P-value comparisons for Figure 1.**

Post-hoc pairwise significance comparisons for auditory testing data using Bonferroni-adjusted t-tests. R does not calculate exact significance values below 2 x 10^-16^. N=18 Foxo3-KO animals and 17 wild-type (WT) animals. Significant values (p<0.05) are highlighted in yellow.
